# Chinese herbal formula Xuefu Zhuyu oral liquid for primary dysmenorrhea: a multicenter randomized controlled trial

**DOI:** 10.3389/fmed.2026.1724529

**Published:** 2026-03-12

**Authors:** Geng Li, Li Zhou, Xin Wang, Shaojun Liao, Wenwei Ouyang, Xiankun Chen, Lixing Cao, Ling Shi, Jie Zhang, Fengjuan Han, Yu Gen, Meiling Xuan, Xiaohui Guo, Zhe Zhang, Zehuai Wen

**Affiliations:** 1 Second Affiliated Hospital of Guangzhou University of Chinese Medicine, Guangzhou, Guangdong, China; 2 Center for Clinical Research, Guangdong Provincial Hospital of Chinese Medicine, Guangzhou, Guangdong, China; 3 Guangdong Provincial Key Laboratory of Clinical Research on Traditional Chinese Medicine Syndrome, Guangzhou, Guangdong, China; 4 State Key Laboratory of Dampness Syndrome of Chinese Medicine, Second Affiliated Hospital of Guangzhou University of Chinese Medicine, Guangzhou, Guangdong, China; 5 Department of Obstetrics and Gynecology, Affiliated Hospital of Liaoning University of Traditional Chinese Medicine, Shenyang, Liaoning, China; 6 Department of Gynecology, Second Affiliated Hospital of Liaoning University of Traditional Chinese Medicine, Shenyang, Liaoning, China; 7 Department of Traditional Chinese Medicine, First Hospital of China Medical University, Shenyang, Liaoning, China; 8 Department of Gynecology, First Affiliated Hospital of Heilongjiang University of Chinese Medicine, Harbin, Heilongjiang, China; 9 Department of Gynecology, Affiliated Hospital of Inner Mongolia University for the Nationalities, Tongliao, Inner Mongolia, China; 10 Innovation Engineering Technology Center for Traditional Chinese Medicine, Liaoning University of Traditional Chinese Medicine, Shenyang, Liaoning, China

**Keywords:** Chinese herbal medicine, placebo, primary dysmenorrhea, randomized controlled trial, Xuefu Zhuyu oral liquid

## Abstract

**Objectives:**

Primary dysmenorrhea (PD), affecting 50%−90% of women, significantly impacts quality of life. However, the need for treatment remains unmet because of side effects and insufficient efficacy. Xuefu Zhuyu (XFZY) oral liquid is a Chinese patent medicine widely used for PD in China, but high-quality evidence is lacking.

**Methods:**

A multicenter double-blind, randomized, placebo-controlled trial was conducted among PD patients aged 18–35 years old. Participants were randomly assigned to receive either XFZY oral liquid or a matched placebo for 3 months. The primary outcome was the change in mean pain intensity as measured by VAS from baseline to the end of treatment. Secondary outcomes included quality of life, the Cox Menstrual Symptom Scale, change of pain duration, painkiller use, etc.

**Results:**

Of 256 eligible participants, 129 received XFZY, and 127 received a placebo. There was no significant difference in the primary outcome between groups (adjusted mean difference: −0.18, 95% CI: −0.67 to 0.31, *P* = 0.463) in FAS analysis. However, the XFZY group had significantly lower rates of painkiller use (9.2% lower during the treatment period, 10.1% lower overall; *P* < 0.05) and consumed fewer painkillers (*P* < 0.05) than the placebo group. Other secondary outcomes were not significantly different between the two groups. Adverse event incidence was similar between groups.

**Conclusions:**

This trial did not demonstrate a benefit of the change in the mean pain intensity in treating PD with XFZY oral liquid. However, XFZY significantly reduced painkiller use and demonstrated good safety, suggesting its potential as an alternative analgesic for PD.

**Clinical trial registration:**

https://www.chictr.org.cn/showproj.html?proj=44287, identifier: ChiCTR1900026819.

## 1 Introduction

Primary dysmenorrhea (PD) is defined as painful menstruation in the absence of pelvic pathology (1, 2). It affects 50%−90% of women, half of whom describe their pain as moderate to severe (2). PD is associated with substantially reduced quality of life, and up to 15% of women experience severe dysmenorrhea symptoms that cause absenteeism from work, school, or other activities (3). It has become a significant health problem for adolescents, that also adversely affects the daily activities of young women (4). Moreover, PD also has an association with an increased risk of depression and anxiety (5).

At present, non-steroidal anti-inflammatory drugs (NSAIDs) and hormonal contraceptives are considered first-line agents for PD (2). However, their potential side effects limit the use of these therapies, such as weight gain reported with the use of depot medroxyprogesterone acetate, gastrointestinal symptoms (1, 6) nephrotoxicity, hematologic abnormalities, and edema caused by NSAIDs (3, 7). Patients may also find them inconvenient because of the periodic, prolonged, or continuous use of hormone therapy (8). Moreover, 20%−25% of patients fail to be relieved of their pain symptoms after being treated with NSAIDs (9). In addition, temporary interventions do not result in permanent cures, as symptoms recur once the medicine is discontinued (10). As a result, some patients seek help with complementary and/or alternative medicine, such as Chinese medicine (CM). For example, researchers found that Honghua Ruyi Pill, a compound herbal medicine, can improve dysmenorrhea symptoms (11).

A popular CM prescription, the Xuefu Zhuyu (XFZY) decoction formula has been widely used in China for centuries (12) for clinical symptoms of PD. XFZY oral liquid is an improved dosage form of the decoction with the same composition. It has been approved by the China Food and Drug Administration (13) and included in Chinese Pharmacopeia (14). The primary components of XFZY are Persicae semen (Taoren), Carthami flos (Honghua) and Rehmanniae radix (Dihuang; see Supplementary File 1). Recently, some studies have found the chemical properties of XFZY (15–18). Although the results of these studies varied slightly, they still provided a reference for the XFZY quality control. XFZY has been known to be associated with enhanced angiogenesis on vascular endothelial cells, inhibition of inflammation and alleviation of oxidative stress (19), which are considered to be related to the pathology and symptoms of dysmenorrhea (20, 21). But the exact mechanism of action of XFZY needs further research. A systematic review has suggested that the XFZY formula may have an effect on PD without serious adverse effects (22). However, due to the low quality of the included studies, the authors concluded that the results of these studies should be interpreted with caution, and randomized trials with rigorous methodological input are needed. Therefore, we conducted this 6-month randomized controlled trial to evaluate the efficacy and safety of XFZY for PD.

## 2 Methods

### 2.1 Study design

This was a multicenter, double-blind, randomized, placebo-controlled trial conducted from May 2020 to August 2022. Ethical approvals were obtained from the ethics committee at Guangdong Provincial Hospital of Chinese Medicine (No. BF2019-175-01) and each center. The study protocol was previously published elsewhere (23) and registered at the Chinese Clinical Trials Registry in 2019 (ChiCTR1900026819). The trial was reported following the CONSORT extension for Chinese herbal medicine formulas (24).

### 2.2 Participants

Eligible participants were women 18–35 years of age with a clinical diagnosis of PD and Qi stagnation and Blood stasis pattern (QBP) in CM (for detailed information on the pattern see Table S1 in Supplementary File 2); who had a menstrual cycle from 21 to 35 days; and had a pain visual analog scale (VAS) score over 4. QBP are common PD patterns. Its features always present as distending pain and stabbing pain. Qi stagnation is a pathological change characterized by impeded circulation of Qi that leads to stagnation of Qi movement and functional disorder of organs, manifested as distention or pain in the affected part. In CM theory, blood stasis is a morbid state of blood stagnancy in a certain area of the body caused by sluggish flow of Qi, eficiency of Qi or Blood (25).

Patients with secondary dysmenorrhea or other severe diseases were excluded from our study. Full eligibility criteria are described in detail in the protocol (23) (also found in Table S2 of Supplementary File 2). Participants with PD were recruited and enrolled from the gynecology clinics at the following six hospitals in China: (1) Affiliated Hospital of Liaoning University of Traditional Chinese Medicine in Liaoning province, (2) Guangdong Provincial Hospital of Chinese Medicine in Guangdong province, (3) Second Affiliated Hospital of Liaoning University of Traditional Chinese Medicine in Liaoning province, (4) First Affiliated Hospital of Heilongjiang University of Chinese Medicine in Heilongjiang province, (5) First Hospital of China Medical University in Liaoning province, and (6) Affiliated Hospital of Inner Mongolia Minzu University in Inner Mongolia. After a maximum 14-day screening period, eligible participants were randomly assigned at a 1:1 ratio to receive either XFZY or a matching placebo. All patients signed written informed consent and were entitled to withdraw from the trial at any time point.

### 2.3 Randomization and blinding

A center-stratified and permuted block randomization sequence was generated by SAS 9.2 software (SAS Institute Inc., Cary, NC, USA), and was performed by the Institute of Basic Research in Clinical Medicine, China Academy of Chinese Medical Sciences (IBRCM; Beijing, China). Eligible participants were randomly allocated to either the XFZY group or the placebo group at a ratio of 1:1 through the Interactive Web Response System developed by IBRCM. The randomization list and blinding codes were kept strictly confidential and only the IBRCM staff had access to them. All investigators, participants, and related medical staff remained unaware of the intervention assignments throughout the trial. Participants were unblinded if they experienced an emergency during the trial, or if a serious adverse event requiring knowledge of the study drug occurred.

### 2.4 Interventions

Participants in the experimental group received XFZY oral liquid orally 20 ml per time, three times a day for three menstrual cycles, with each cycle lasting 14 days before the onset of menstruation. After treatment, there was a 12-week follow-up.

Participants in the control group received a placebo without any pharmacodynamic ingredients, but which was identical to XFZY oral liquid in dosage, color, appearance, smell, taste, texture, and packaging. The placebo consisted of honey, white granulated sugar, fried brown sugar, bitterant, ginseng essence, natural edible pigments, and food antiseptic. All other treatment settings were the same as in the experimental group (see Supplementary File 1 for more information).

The XFZY oral liquid and the placebo were manufactured by Jilin Aodong Yanbian Pharmaceutical Co. Ltd. (Jilin, China) according to the requirements of good manufacturing practice (GMP). A previous study has shown that paeoniflorin, naringin, glycyrrhizic acid, ferulic acid, and hydroxysafflor yellow A can be used for the quality control of XFZY oral liquid (15). Therefore, ultra-high performance liquid chromatography was used to tackle the quality control of XFZY oral liquid, and it showed that the feature peak paeoniflorin and naringin were detected in the figure print map (Supplementary File 1). Both XFZY and the placebo were labeled and packaged to meet blinding requirements by Jilin Aodong Yanbian Pharmaceutical Co. Ltd. under the supervision of IBRCM.

All participants were advised not to take any other treatments for PD during the trial. Painkillers (fendid, a form of ibuprofen sustained-release capsule) was allowed as a rescue treatment in case patients underwent unbearable pain during menstruation. Other drugs that alleviate menstrual pain were prohibited, such as oral contraceptives. Chinese herbal medicines that have the effect of moving Qi and activating Blood were also prohibited.

### 2.5 Outcome measurements

The primary outcome is a change in the mean pain intensity, as measured by the visual analog scale (VAS), from baseline to the end of treatment. The secondary outcomes included the change in menstrual pain duration, the change in peak pain intensity as measured by the VAS, CM syndrome change, Cox Menstrual Symptom Scale (CMSS), quality of life measured by EQ-5D-5L, and painkiller consumption.

### 2.6 Safety assessment

Adverse events (AEs) experienced by participants at any point in the trial were recorded, and any abnormal laboratory findings with clinical significance were also recorded as AEs. Verbatim AEs were coded using Medical Dictionary for Regulatory Activities (MedDRA) (26). Investigators assessed the causality between AEs and interventions following the WHO-UMC causality assessment system (27).

### 2.7 Data collection and management

An independent team from Jilin Aodong Yanbian Pharmaceutical Co. Ltd. was established to monitor the collection and management of data for the trial under the auditing of IBRCM in accordance with a developed data management plan to ensure data quality. All participants were asked to complete a dysmenorrhea diary to record the time of menstrual onset, duration, daily pain level (assessed by VAS), start and end time of each painful episode, highest pain level of the present menstrual cycle, and use of any medication (including rescue and treatment medication). Study visits occurred at screening, at baseline (1–3 days after first menstruation), and at 1–3 days after the second, third, and fourth menstruations. Then, follow-up visits at 1–3 days after the fifth, sixth, and seventh menstruations were conducted (23).

Participants self-rated their mean and peak pain intensity using VAS, reported menstrual pain duration, and filled out the CMSS at the baseline and at each visit. EQ-5D-5L was completed by each participant at the baseline, the end of treatment, and the end of the follow-up period. Investigators assessed the subjects' CM syndrome at the baseline and at each visit, as well as health economics at the baseline and at each visit during the treatment duration. Additionally, investigators confirmed the painkiller consumption and pain duration at every visit by checking the diaries.

### 2.8 Statistical analysis

In this superiority trial, the mean changes in VAS score (noted as “mean ± standard deviation”) after 12 weeks of treatment (three menstrual cycles) for the XFZY group and the placebo group were assumed to be 3.00 ± 0.94 and 0.88 ± 1.64, respectively (23). The minimal clinically important difference (MCID) for VAS was 1.6 for acute abdominal pain (28). Therefore, considering a superiority margin of 1.6 and a 15% loss to follow-up, we calculated that the sample size of 124 for each group could provide 80% power at a one-sided significance level of 2.5% in the trial.

The data were processed with PASW Statistics 18.0 (IBM SPSS Inc., Armonk, NY, USA) and SAS 9.4 (SAS Institute Inc., Cary, NC, USA). Two-tailed *P* < 0.05 was considered statistically significant. The analysis was based on the intent-to-treat (ITT) and per-protocol (PP) principles, respectively. The full analysis set (FAS) included all participants who were randomized and had at least one visit record. Missing data were imputed using the multiple imputation method. The PP population only included participants without major protocol deviations or low compliance.

Demographic and other baseline data were presented by descriptive statistics. The primary outcome measure was presented as a mean and standard deviation (SD) alongside adjusted mean differences with corresponding 95% confidence intervals (CIs) derived from a generalized linear model. Mean differences (MD) were adjusted for the center and for baseline pain VAS score for the past 6 months. Pain VAS scores were further analyzed using a repeated-measure analysis model which included all assessment times.

For secondary outcomes, continuous and categorical data were compared between the two groups with either a *t*-test, Chi-square test or Fisher's exact test. Frequency, mean, SD, median, and the range of outcome variables were summarized. To analyze the factors affecting the outcome change at each time point, either a mixed-effects, linear, or logistic regression model was performed to adjust for baseline characteristics such as age, menstrual pain severity, and other covariables. Frequency differences for AEs and adverse drug reactions were compared with a Chi-square test or Fisher's exact test.

Pre-specified subgroup analyses were performed based on disease severity (a VAS score for peak pain intensity of either less than 7, or greater than or equal to 7). Exploratory subgroup analysis was conducted based on the use of painkillers (yes or no).

## 3 Results

### 3.1 Baseline characteristics

**Figure 1:**
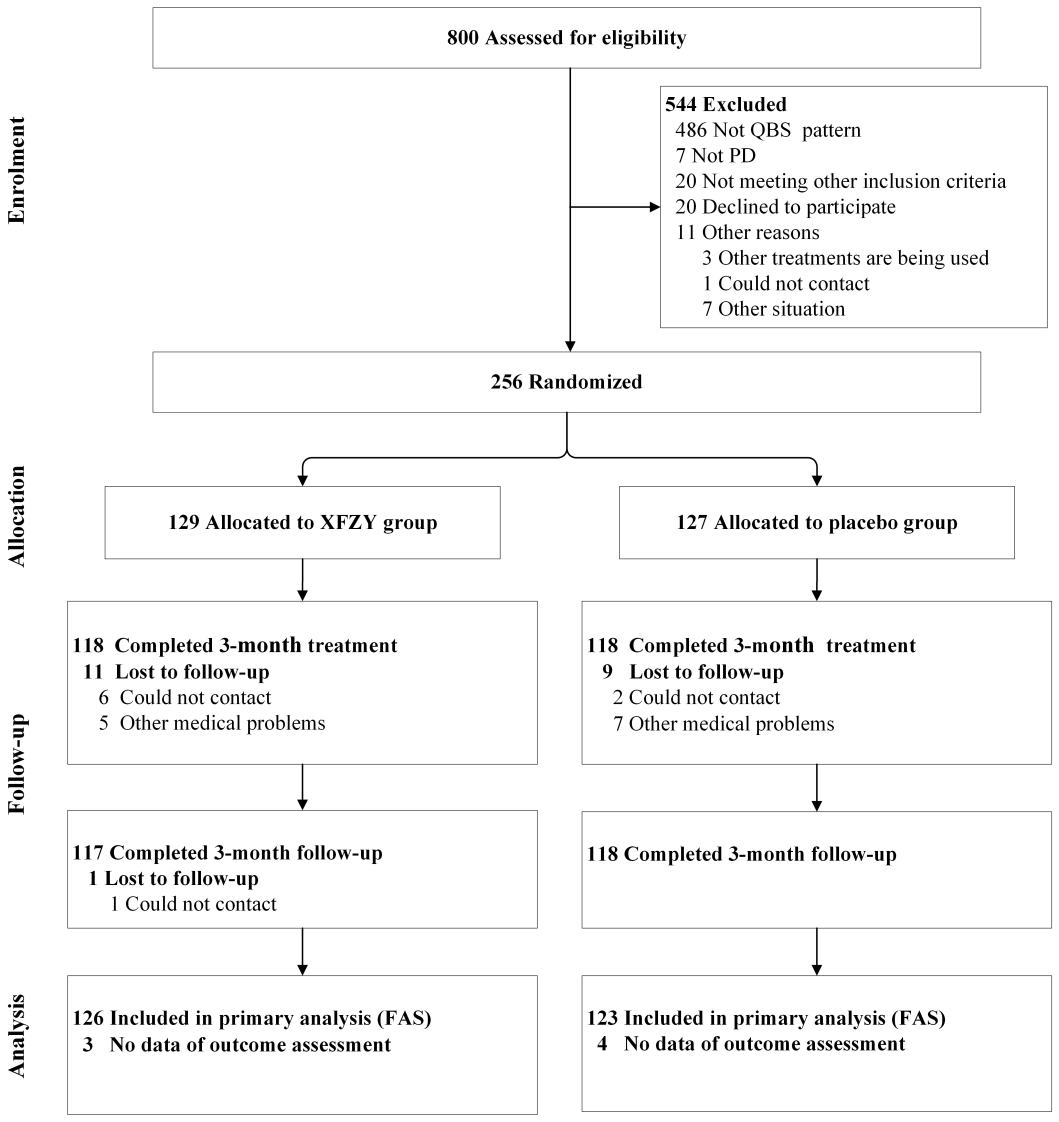
Study flow chart. PD, primary dysmenorrhea; XFZY, Xuefu Zhuyu oral liquid; FAS, full analysis set.

A total of 800 potential patients were screened from July 2020 to August 2022, of whom 544 were ineligible or declined to participate. Ultimately, 256 were enrolled and randomly assigned (Figure 1). The groups were well-balanced in terms of all characteristics measured at the baseline (Table 1).

**Table 1:** Participants' demographics and baseline characteristics.

**Characteristics**	**XFZY group (*n* = 126)**	**Placebo group (*n* = 123)**
Age (SD), years	24.80 (2.94)	25.09 (3.13)
**Nationality (%)**		
Han	102 (81.0)	107 (87.0)
Other	24 (19.0)	16 (13.0)
**Marital status (%)**		
Married	10 (7.9)	11 (8.9)
Single	116 (92.1)	112 (91.1)
**Education (%)**		
Secondary	0 (0.0)	3 (2.4)
Tertiary	62 (49.2)	57 (46.3)
Master's or above	64 (50.8)	63 (51.2)
**Occupation (%)**		
Manual worker	0 (0.0)	2 (1.6)
Non-manual worker	126 (100.0)	121 (98.4)
Non-smoker (%)	126 (100.0)	123 (100.0)
Non-drinker (%)	126 (100.0)	123 (100.0)
Course of disease (SD), months	70.52 (44.75)	70.24 (42.50)
Mean pain VAS (SD)	5.74 (1.25)	5.95 (1.33)
Highest pain VAS (SD)	6.96 (1.25)	7.00 (1.24)
Days unable to participate in regular activities for the past 6 months (SD)	4.75 (4.18)	4.12 (4.35)
Mean pain VAS score for past 6 months (SD)	6.77 (1.23)	6.74 (1.29)
**PD severity (%)**		
Mean VAS < 7	102 (81.0)	96 (78.0)
Mean VAS ≥7	24 (19.0)	27 (22.0)
**PD severity (%)**		
Highest VAS < 7	64 (50.8)	60 (48.8)
Highest VAS ≥7	62 (49.2)	63 (51.2)
**Previous treatment (%)**		
No	81 (64.3)	78 (63.4)
Yes	45 (35.7)	45 (36.6)
**Allergies (%)**		
No	108 (85.7)	99 (80.5)
Yes	18 (14.3)	24 (19.5)
**Regular menstrual cycle (%)**		
No	6 (4.8)	5 (4.1)
Yes	120 (95.2)	118 (95.9)
Time of menarche (SD) years	13.12 (1.24)	13.24 (1.02)
Menstrual cycle (SD) days	29.90 (2.32)	29.61 (2.22)
Length of menstrual period (short) (SD)	4.98 (1.04)	4.82 (1.06)
Length of menstrual period (long) (SD)	6.44 (1.04)	6.33 (1.02)
No	24 (19.0)	28 (22.8)
Yes	102 (81.0)	95 (77.2)
TCM syndrome scale (SD)	11.73 (2.88)	11.76 (3.24)
Duration of dysmenorrhea at enrollment (SD), hours	23.91 (20.20)	24.23 (26.14)

XFZY, Xuefu Zhuyu oral liquid; VAS, visual analog scale; SD, standard deviation, VAS, visual analog scale.

### 3.2 Primary outcome

There were no significant differences in the change in VAS scores in mean pain intensity between groups at the end of treatment in FAS (Table 2). From baseline to 3-month, the VAS score for mean pain intensity decreased 2.78 (SD: 2.06) in the XFZY group, compared to a decrease of 2.60 (SD: 2.22) in the placebo group (adjusted MD: −0.18, 95% CI: −0.67 to 0.31, *P* = 0.463), while analysis based on the PP population showed that the differences between groups were significant (adjusted MD: −0.83, 95% CI: −1.37 to −0.29, *P* = 0.003) in the generalized linear model with adjusting for the center and a baseline mean pain VAS score for the past 6 months (Table S3 in Supplementary File 2). Thus, the reduction of 0.83 did not reach the superiority margin of 1.6. Moreover, among those who did not use painkillers, the subgroup analysis showed that the VAS change score in the XFZY group decreased by 0.58 points more than the placebo group at the 1–3 months periods (Table 3).

**Table 2:** Primary outcome assessment.

**VAS score for mean pain intensity**	**XFZY group (*n* = 126) (mean ±SD)**	**Placebo group (*n* = 123) (mean ±SD)**	**Adjust mean difference (95% CI)^a^**	***P-*value**
3-month	2.96 ± 1.93	3.35 ± 2.03	−0.39 (−0.86, 0.08)	0.104
Change in VAS from baseline to 3-month	−2.78 ± 2.06	−2.60 ± 2.22	−0.18 (−0.67, 0.31)	0.463

VAS, visual analog scale; XFZY, Xuefu Zhuyu oral liquid; SD, standard deviation; CI, confidence interval.

^a^The analysis used a generalized linear model; adjusted for the center and for baseline mean pain VAS score for the past 6 months.

**Table 3:** Analysis of painkiller consumption.

**Outcomes**	**XFZY (*n* = 126)**	**Placebo (*n* = 123)**	**95% CI of Difference**	***P-*value**
**Number of participants using painkiller**				
Baseline to 3-month (%)	13 (10.3)	24 (19.5)	−9.2 (−18.0, −0.4)^*^	0.041^a^
Baseline to 6-month (%)	16 (12.7)	28 (22.8)	−10.1 (−19.5, −0.7)^*^	0.037^a^
**Cumulative painkiller consumption (mg), Median (10%, 90%)**				
Baseline to 3-month	0 (0, 90)	0 (0, 900)	/	0.019^b^
Baseline to 6-month	0 (0, 300)	0 (0, 1200)	/	0.020^b^
**Subgroup analysis (whether or not using painkiller) for VAS change**				
Baseline to 3-month				
Not using painkiller	113	99	−0.58 (−1.11, −0.04)	0.034^c^
Using painkiller	13	24	1.99 (0.03, 3.95)	0.047^c^
Baseline to 6-month				
Not using painkiller	110	95	−0.52 (−1.06, 0.01)	0.055^c^
Using painkiller	16	28	1.18 (−0.61, 2.97)	0.190^c^

VAS, visual analog scale; XFZY, Xuefu Zhuyu oral liquid; CI, confidence interval.

^*^Rate of painkiller used (%) and 95% CI.

^a^Chi-square test was used.

^b^Mann-Whitney test was used.

^c^t-test was used.

**Figure 2:**
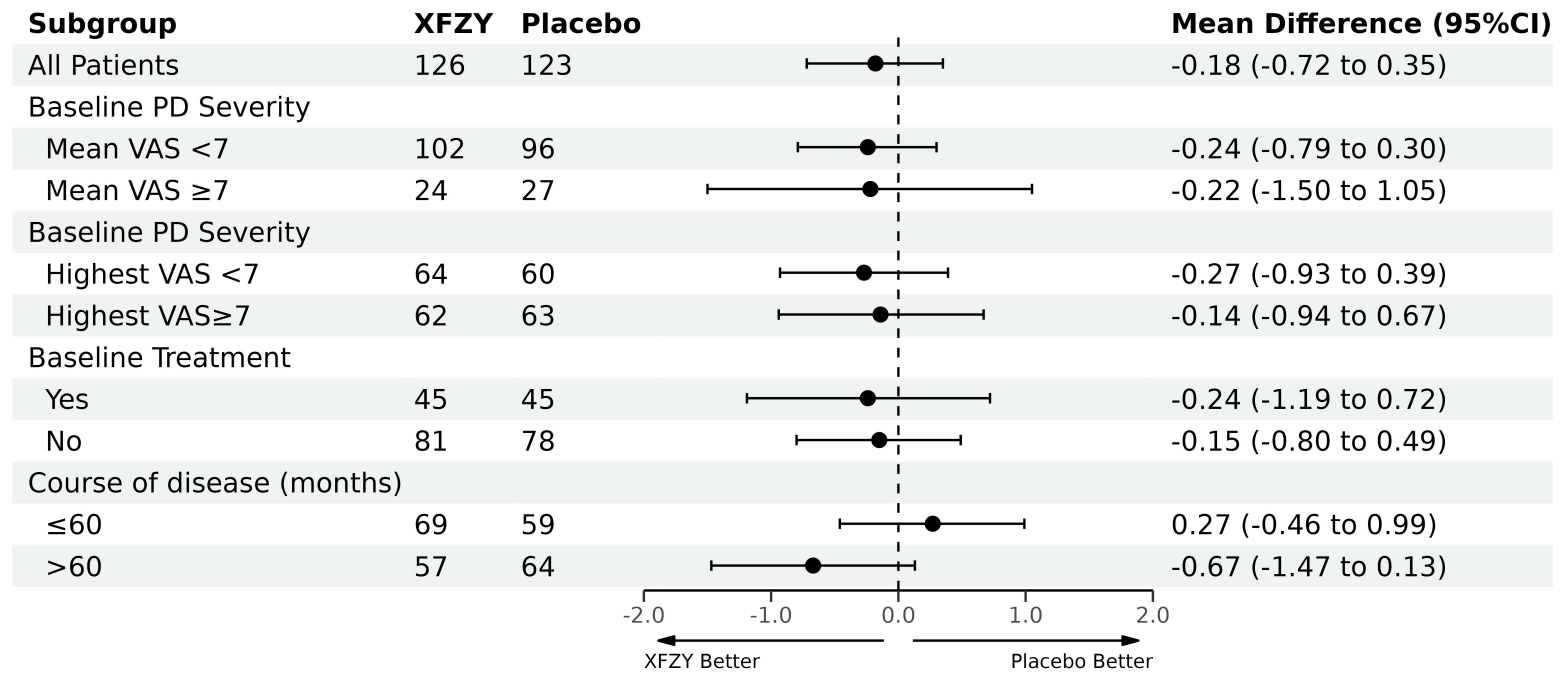
Subgroup analysis. XFZY, Xuefu Zhuyu oral liquid; VAS, visual analog scale; CI, confidence interval; *t*-test was used.

Point estimates and 95% CIs (Table S4 in Supplementary File 2) and further analyses from the repeated-measures model were consistent with the primary analysis (Figure S1 in Supplementary File 2). Furthermore, there was no evidence of varying effects in the pre-specified subgroup analyses (Figure 2 and Table S5 in Supplementary File 2).

### 3.3 Secondary outcomes

The number of women who took painkillers between the XFZY and placebo groups was significantly different; the placebo group had a 9.2 and 10.1% higher rate of painkiller use than the XFZY group during the 1–3 month and 1–6 month periods, respectively (*P* < 0.05; Table 3). What is more, the cumulative painkiller consumption between groups was significantly different; that is, participants in the placebo group taking more painkillers (Table 3). No significant differences were noted in the VAS score reduction in peak pain intensity from baseline to 3 and 6 months or in any other patient-reported secondary outcomes based on the FAS (Table 4). There was also a significant difference between the two groups in the proportion of CM syndrome that had changed by the end of the follow-up period (Table S6 in Supplementary File 2).

**Table 4:** Secondary outcomes assessments.

**Secondary outcome measures**	**XFZY group (*n* = 126)**	**Placebo group (*n* = 123)**	**Mean difference (95% CI)**	***P*-value**
**Change in VAS (peak pain intensity), Mean** ±**SD**				
3-month	−2.95 ± 2.34	−2.60 ± 2.32	−0.35 (−0.95, 0.25)	0.250
6-month	−3.15 ± 2.65	−3.24 ± 2.51	−0.10 (−0.76, 0.56)	0.769
**Pain duration (Hours), Mean** ±**SD**				
3-month	−12.18 ± 18.84	−12.51 ± 20.49	0.32 (−4.72, 5.37)	0.899
6-month	−14.87 ± 18.52	−14.03 ± 23.66	−0.84 (−6.30, 4.63)	0.764
**CMSS total score, Mean** ±**SD**				
3-month	−15.10 ± 14.33	−12.52 ± 14.40	−2.58 (−6.28, 1.12)	0.171
6-month	−18.34 ± 15.40	−16.49 ± 13.87	−1.85 (−5.62, 1.92)	0.334
**CMSS duration score, Mean** ±**SD**				
3-month	−7.93 ± 7.80	−6.76 ± 8.53	−1.17 (−3.28, 0.93)	0.274
6-month	−9.69 ± 8.36	−8.69 ± 8.09	−1.00 (−3.12, 1.12)	0.352
**CMSS severity score, Mean** ±**SD**				
3-month	−7.17 ± 7.01	−5.76 ± 6.66	−1.41 (−3.17, 0.35)	0.116
6-month	−8.66 ± 7.40	−7.81 ±.67	−0.85 (−2.66, 0.96)	0.357
**EQ-5D-5L, Mean** ±**SD**				
3-month	0.01 ± 0.08	0.01 ± 0.05	−0.00 (−0.02, 0.02)	0.838
6-month	0.01 ± 0.07	0.02 ± 0.07	−0.00 (−0.02, 0.01)	0.662
**EQ-5D-5L VAS, Mean** ±**SD**				
3-month	2.85 ± 7.24	1.95 ± 8.62	0.90 (−1.15, 2.95)	0.390
6-month	2.94 ± 9.61	4.93 ± 14.61	−1.99 (−5.23, 1.24)	0.226

XFZY, Xuefu zhuyu oral liquid; VAS, visual analog scale; CI, confidence interval; SD, standard deviation; EQ-5D-5L, EQ-5D 5 lever version; t-test was used.

### 3.4 Safety assessments

Ten AEs in the XFZY group and four in the placebo group were reported (Table 5 and Table S7 in Supplementary File 2). One participant in the XFZY group complained of constipation, and the researcher judged that it may have been related to XFZY. All AEs were mild or moderate. No serious AEs occurred. Other than one participant in each group who used a painkiller for headaches, no patients experienced AEs that required medical interventions.

**Table 5:** Safety analysis.

**Safety outcomes**	**XFZY group (*n* = 129)**	**Placebo group (*n* = 126)**	***P-*value**
Number of patients (%)	7 (12.6)^a^	3 (11.5)^b^	0.334^c^
Number of AEs	10	4	**/**

^a^One patient experienced 3 AEs, two headaches, and one episode of diarrhea; another patient experienced 2 AEs, constipation and sore throat. ^b^One patient experienced 2 AEs, diarrhea and abdominal distension. ^c^Fisher's exact test was used.

## 4 Discussions

This multicenter, double-blind, randomized, placebo-controlled trial showed that XFZY oral liquid was not superior to placebo in reducing mean pain in PD patients in the full analysis population. However, we found that the adjusted MD between groups was significant (−0.83, 95% CI: −1.37 to −0.29) in the PP population after adjusting for the confounding factors. In addition, the number of painkillers used in the XFZY group decreased by 9.2 and 10.1% during the 1–3 month and 1–6 month periods, respectively, compared with the placebo group. Meanwhile, the cumulative painkiller consumption between groups was significantly different; that is, participants in the XFZY group were taking fewer painkillers (Table 3). It partly demonstrated the efficacy of XFZY oral liquid. Meanwhile, the incidence of AEs was low and similar in both groups.

Painkillers are widely used in dysmenorrhea patients (29, 30). In our study, PD participants were allowed to use painkillers (ibuprofen) when they could not tolerate the pain. Painkiller consumption was used as a secondary outcome to reflect the indirect effect of XFZY by measuring the amount of analgesics used in the two groups. We found that the XFZY group had a lower proportion of painkiller use and less cumulative painkiller consumption than the placebo group. The reduced painkiller consumption suggests that XFZY may act as an alternative pain reliever. Thus, this result reveals that XFZY exerts its effects by reducing the consumption of painkillers. From one side, a subgroup analysis found that the pain VAS changes in the XFZY group decreased significantly more than the placebo group in the participants who did not use painkillers at the 1–3 months periods (Table 3). Given the side effects of NSAIDs, such as edema (3, 7), increased risks of heart failure, elevated blood pressure, and an increased risk of thrombotic events (31), patients with PD using XFZY may reduce the use of NSAIDs, has also to some extent decreased the occurrence of these side effects.

One important pathophysiology of PD is increased prostanoid secretion by way of the cyclooxygenase pathway. The major culprits involved in PD are PGF2a and PGE2, which are two types of prostaglandins (PGs) (3). A review has summarized that the underlying mechanism of herbal medicine for PD is associated with PG level reduction, suppression of cyclooxygenase-2 expression, superoxide dismutase activation and malondialdehyde reduction, nitric oxide, inducible nitric oxide synthase and nuclear factor-kappa B reduction, stimulation of somatostatin receptor, intracellular Ca2+ reduction, and the recovery of phospholipid metabolism (32). Several studies have also shown that XFZY can raise PGE2 levels and lower PGF2α levels in patients with PD (33, 34). The studies mentioned above partially explain the analgesic effect of XFZY oral liquid. But exploring the mechanism of XFZY on primary dysmenorrhea is till insufficient, and it need to be further studied in the future.

The results of this trial in FAS analysis contradict the findings of a previous systematic review (22) which found that the XFZY combined with Western medicine had a significantly lower VAS score than Western medicine therapy alone. This may have been due to the following considerations. Firstly, the intervention in our study differed from that in the systematic review. Both trials included in the systematic review had add-on designs, and the intervention was based on routine medicine therapy. Secondly, the mean difference in VAS score in the systematic review was low (−0.51, 95% CI: −0.59, −0.43), which is significantly less than the MCID for acute pain (23). Thirdly, one of the trials included in the systematic review may have had errors in the VAS data (22). Finally, in our study, the painkiller consumption was less in the XFZY group compared with placebo, indicating an alternative analgesic effect of XFZY oral liquid. and when we excluded participants who had taken painkillers, we found that the XFZY group had a greater decrease in VAS score than the placebo group.

### 4.1 Limitations

Several limitations of this study should be considered. Firstly, participants' enrollment and follow-up were affected by the COVID-19 pandemic. This may have influenced the efficacy assessment of XFZY oral liquid. Secondly, this trial's treatment regimen entailed that participants take XFZY oral liquid or placebo for 14 days before the onset of menstruation. However, the participants' menstrual cycles ranged from 21 to 35 days, and some of their cycles varied in length. Thus, it was difficult for them to know when to start treatment. This may present another defect of this study, and may have influenced the efficacy assessment of XFZY oral liquid.

## 5 Conclusions

This trial demonstrated that XFZY oral liquid was not beneficial for PD patients in reducing VAS pain scores, compared with placebo in FAS analysis. However, fewer PD patients in the XFZY group took painkillers than in the placebo group, with good tolerance of safety assessment. Furthermore, the reduced painkiller consumption in the XFZY group suggests that XFZY oral liquid may act as an alternative pain reliever for PD.

## Data Availability

The original contributions presented in the study are included in the article/Supplementary material, further inquiries can be directed to the corresponding author.
