# Supplementary material for: Chinese herbal formula Xuefu Zhuyu oral liquid for primary dysmenorrhea: a multicenter randomized controlled trial
Source: Front Med (Lausanne). 2026 Mar 12;13:1724529. doi: 10.3389/fmed.2026.1724529 (PMC13017913; doi:10.3389/fmed.2026.1724529)
Supplement: Supplementary file 1 [file Data_Sheet_1.docx]

Chinese Herbal Formula Xuefu Zhuyu Oral Liquid for Primary Dysmenorrhea: A Multicenter Randomized Controlled Trial

Geng Li ^1,2,3,4^, Li Zhou ^1,2^, Xin Wang ^5^, Shaojun Liao ^1^, Wenwei Ouyang ^1,2^, Xiankun Chen ^1,2,3,4^, Lixing Cao ^1,2,3,4^, Ling Shi ^6^, Jie Zhang ^7^, Fengjuan Han ^8^, Yu Gen ^9^, Meiling Xuan ^1,2^, Xiaohui Guo ^1,2^, Zhe Zhang ^10^, Zehuai Wen^1,2,3,4^ ^*^

# The composition and quality control standards of XFZY oral liquid

1. XFZY is processed from the Xuefu Zhuyu Decoction formula in the *Corrections on the Errors of Medical Works* (Yilin Gaicuo) by Wang Qingren of the Qing Dynasty. It is composed of 11 herbs: *peach kernel* (Taoren), *safflower* (Honghua), *rehmannia glutinosa* (Dihuang), *angelica sinensis* (Dangui), *ligusticum chuanxiong* (Chuangxiong), *red peony root* (chishao), *radix achyranthis bidentatae* (Niuxi), *radix platycodonis* (Jiegeng), *radix bupleurum* (Chaihu), *bran fried fructus aurantia* (Zhiqiao), and *licorice* (Gancao). It has the effect of activating blood and resolving stasis, moving qi to relieve pain.^[1]^

In the formula, taoren is effective in breaking blood and expelling stasis, and safflower can move stagnation and activate blood and resolve stasis; they are both sovereign medicinal. Dangui nourishes and activates blood, and moves qi to dispels stasis without health damage. Chaihu is effective in soothing the liver to release depression, regulating qi to dissipate binds. Jiegeng can diffuse the qi from depression, and drug-load upward. Zhiqiao moves qi to remove stagnation, removes distention and diffuses stuffiness. Dangui, chaihu, jiegeng and zhiqiao are all minister medicinal. Chishao activates blood to relieve pain. Niuxi dispels stasis and conducts blood downward. Shengdi clears heat to cool blood. Chishao, niuxi and shengdi are all assistant medicinal. Gancao harmonizes all medicinal, and it is a courier medicinal.

XFZY oral liquid is an improved dosage form of XFZY decoction. It strictly follows the original prescription of XFZY decoction with the use of high-tech means to retain the active ingredients of the drug to the maximum extent. As such, it is a veritable concentrated XFZY decoction.[2]

Table S1 The dose and scientific names of all ingredients in XFZY oral liquid

| **Chinese name** | **English name** | **Latin name** | **Dose*** |
| --- | --- | --- | --- |
| Chaihu | Bupleuri Radix | *Bupleurum chinense DC.* | 17 g |
| Danggui | Angelicae Sinensis Radix | *Angelica sinensis (Oliv.) Diels* | 50 g |
| Shengdihuang | Rehmanniae Radix | *Rehmannia glutinosa Libosch.* | 50 g |
| Chishao | Paeoniae Radix Rubra | *Paeonia lactiflora Pall* | 33 g |
| Honghua | Carthami Flos | *Carthamus tinctorius L.* | 50 g |
| Taoren | Persicae Semen | *Prunus persica (L.) Batsch* | 67 g |
| Zhiqiao | Aurantii Fruxtus | *Citrus aurantium L.* | 33 g |
| Gancao | Glycyrrhizae Radix et Rhizoma | *Glycyrrhiza uralensis Fisch.* | 17 g |
| Chuanxiong | Chuangxiong Rhizoma | *Ligusticum chuanxiong Hort.* | 25 g |
| Niuxi | Achyranthis Bidentatae Radix | *Achyranthes bidentata Bl.* | 50 g |
| Jiegeng | Platycodonis Radix | *Platycodon grandiflorum (Jacq.) A.DC.* | 25 g |

*The dose of each drug in 1,000 ml liquid

**2. Pharmaceutical production processes for XFZY oral liquid**:

XFZY oral liquid was manufactured in strict accordance with the standards of *Chinese Pharmacopoeia* (2020), and the process strictly abided by the good manufacturing practices (GMP). The outline is as follows:

*Bupleuri radix, angelicae sinensis radix, aurantii fruxtus, and chuangxiong rhizoma* were distilled to extract aromatic water, and set aside; medicine residue and other herbal medicines were decocted 3 times, 2 hours each time. The decoction was combined and filtered, and the filtrate was concentrated to a relative density of about 1.10 (60°C). Ethanol was added such that the alcohol content constituted 60%. Then, it was refrigerated for 24 hours, filtered, and the ethanol filtrate was recycled until there was no alcohol smell. Next, 100 g of sucrose, 200 g of honey and 0.5 g of potassium sorbate were added to the above aroma water. It was then stirred, and water was added until there was 1,000 ml and it was mixed well. The pH was adjusted to 5.0, and then it was refrigerated, filtered, packaged, sterilized, and prepared.

**3. According to the 2020 edition of *Chinese Pharmacopoeia,*^[3]^ the quality control standards for the XFZY oral liquid are:**

1. Thin layer chromatography (TLC) is used to identify radix bupleurum (chaihu), bran fried fructus aurantia (zhiqiao), licorice (gancao), angelica sinensis (dangui), ligusticum chuanxiong (chuangxiong), radix achyranthis bidentatae (niuxi) and radix platycodonis (jiegeng).
2. Content determination: Each 1 ml containing red peony root (chishao) is calculated by paeoniflorin (C23H28O11), and is no less than 0.25 mg; bran fried fructus aurantia (zhiqiao) is calculated by naringin (C27H32O14), and is no less than 0.66 mg; peach kernel (taoren) is calculated by amygdalin (C20H27NO11), and is no less than 0.67 mg. The test solution and control solution are analyzed by ultra-high performance liquid chromatography (UHPLC) (Fig 1).


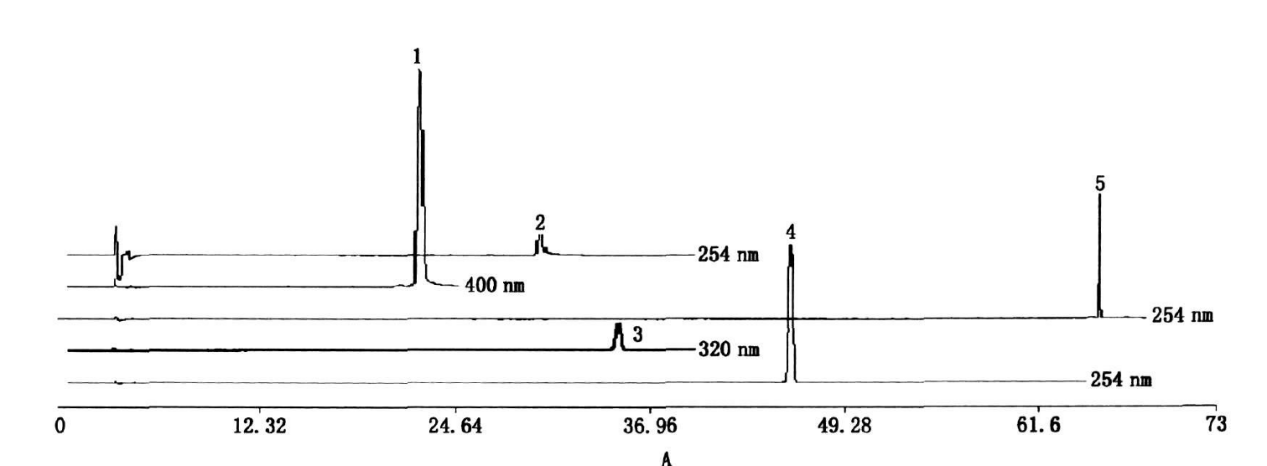


Fig 1. UHPLC chromatogram

Note: 1. hydroxysafflor yellow A, 2.ferulic acid, 3. paeoniflorin, 4.naringin, 5.glycyrrhizic acid

1. **Dosage and scientific names of all ingredients in the placebo**

| **English name** | **Dose^#^** |
| --- | --- |
| Honey | 100 g |
| White granulated sugar | 200 g |
| Fried white sugar | 19 g |
| Fried brown sugar | 7 g |
| Bitterant | 0.05 g |
| Potassium sorbate | 1 g |
| Sodium benzoate | 1 g |
| Ethanol (95%) | 50 ml |
| Ginseng essence | 0.1 g |

^#^The drugs above are produced in 1,000 g finished products.

References

[1] Huang Zhidong, Wang Xiaoling. Clinical application of Xuefu Zhuyu Decoction in gynecology of traditional Chinese Medicine. Cardiovascular Disease Electronic Journal of Integrated Traditional Chinese and Western Medicine.2018;6(18):164-165.

[2] Guangyao Wang. Xuefu Zhuyu Oral Liquid, a classic drug for activating blood and removing blood stasis[J]. Jilin Journal of Traditional Chinese Medicine. 2004; 24(08):62.

[3] Chinese Pharmacopoeia commission. The Pharmacopoeia of the People's Republic of China 2020 Edition. China Medical Science and Technology Press, 2020:894
