# Supplementary material for: Chinese herbal formula Xuefu Zhuyu oral liquid for primary dysmenorrhea: a multicenter randomized controlled trial
Source: Front Med (Lausanne). 2026 Mar 12;13:1724529. doi: 10.3389/fmed.2026.1724529 (PMC13017913; doi:10.3389/fmed.2026.1724529)
Supplement: Supplementary file 2 [file Data_Sheet_2.docx]

Chinese Herbal Formula Xuefu Zhuyu Oral Liquid for Primary Dysmenorrhea: A Multicenter Randomized Controlled Trial

Geng Li ^1,2,3,4^, Li Zhou ^1,2^, Xin Wang ^5^, Shaojun Liao ^1^, Wenwei Ouyang ^1,2^, Xiankun Chen ^1,2,3,4^, Lixing Cao ^1,2,3,4^, Ling Shi ^6^, Jie Zhang ^7^, Fengjuan Han ^8^, Yu Gen ^9^, Meiling Xuan ^1,2^, Xiaohui Guo ^1,2^, Zhe Zhang ^10^, Zehuai Wen^1,2,3,4^ ^*^

1. **Supplementary Figures and Tables**
   1. **Supplementary Figures**


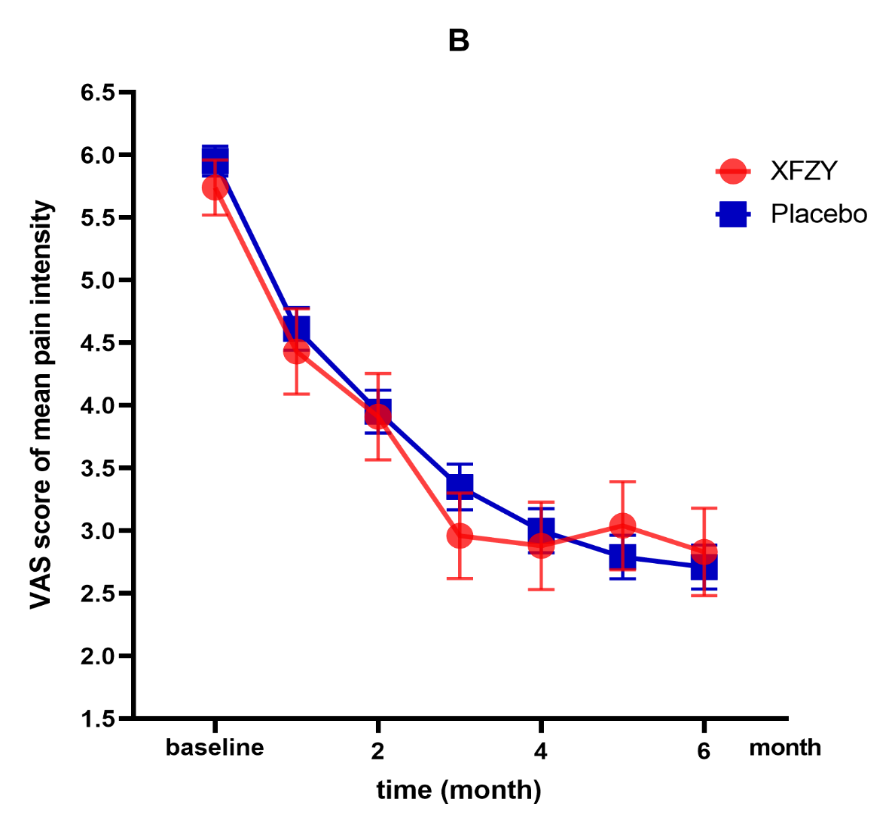


**Fig S1. VAS score of mean pain intensity** (repeated-measures model was used; VAS: visual analogue scale; XFZY: Xuefu Zhuyu oral liquid).

- 1. **Supplementary Tables**

**Table S1 CM syndrome diagnostic scale for QBP ^[1]^**

| **Symptoms/signs** | **Yes** | **No** | **Score** |
| --- | --- | --- | --- |
| Pain | 9 | 0 |  |
| Irritability/depression | 16 | 0 |  |
| Distending pain | 2 | 0 |  |
| Scurry pain | 6 | 0 |  |
| Chest distress | 0.5 | 0 |  |
| Lumps in body | 7 | 0 |  |
| Petechia in the tongue | 4 | 0 |  |
| Purplish tongue | 1 | 0 |  |
| Unsmooth pulse | 4 | 0 |  |
| Deep pulse | 2 | 0 |  |
| Total score  If ≥ 20 points, it is diagnosed as QBP | | |  |

**Notes: Pains include stomachache, abdominal pain, low back pain, dysmenorrhea, breast pain, limb pain, etc.** CM: Chinese medicine, QBP: Qi-Stagnation and Blood-Stasis pattern. [1] Wang J, An Y, Li ZL, et al. Development of PRO scale for qi stagnation and blood stasis syndrome. Chinese J Exp Tradit Med Formulae, 2018, 24(15): 16-20.

**Table S2 inclusion and exclusion criteria**

| **Inclusion criteria** | 1. Meet the diagnostic criteria for PD, and QBP in CM  2. Aged 18 to 35 years  3. Menstrual cycle (28 + 7) days  4. Pain visual analogue scale (VAS) score > 4  5. Signed informed consent |
| --- | --- |
| **Exclusion criteria** | 1. Secondary dysmenorrhea confirmed by gynecological ultrasound or caused by pelvic inflammation, endometriosis, cervix tumor, endometrial polyp, or other ailments  2. Severe primary cardiovascular, liver, kidney or blood disease, mental illness (schizophrenia, epilepsy, alcoholism, anorexia, and/or a history of serious mental illness, and those taking antidepressants, antiserotonin, barbiturates or psychotropic drugs);  3. Lactating and pregnant women or women who have recently been preparing for pregnancy  4. Allergies to the drug ingredients in the study  5. Participants in other clinical trials  6. Those who have been treated with hormone drugs over the last 3 months  7. Self-Rating Anxiety Scale (SAS) ≥ 60 or Self-Rating Depression Scale (SDS) > 62. |

**Table S3 Comparison of primary outcome between treatment groups base on PPS**

| **VAS score for mean pain intensity** | **XFZY group**  **(n=81)**  **(mean±SD)** | **Placebo group**  **(n=102)**  **(mean±SD)** | **Adjust mean difference ***  **(95% CI)** | ***P*** |
| --- | --- | --- | --- | --- |
| **3-month** | 2.42±1.72 | 3.34±1.97 | -0.93 (-1.43, -0.42) | 0.000 |
| **Change in VAS from baseline to 3-month** | -3.48±1.88 | -2.66±2.21 | -0.83 (-1.37, -0.29) | 0.003 |

Missing data were imputed by multiple imputation method.

*Adjusted for the center and for baseline pain VAS score for the past 6 months.

PPS: per-protocol set; XFZY: Xuefu Zhuyu oral liquid; VAS: visual analogue scale; SD: standard deviation; CI: confidence interval.

**Table S4 Comparison of primary outcome between treatment groups**

| **VAS for mean pain** | **XFZY group**  **(n = 126)** | **Placebo group**  **(n = 123)** | **Mean difference (95% *CI*)** | ***P*** |
| --- | --- | --- | --- | --- |
| **Baseline** | 5.74±1.25 | 5.95±1.33 | -0.21(-0.53,0.11) | 0.201 |
| **1-month** | 4.43±1.94 | 4.61±1.89 | -0.19(-0.66,0.29) | 0.447 |
| **2-month** | 3.91±1.96 | 3.95±1.90 | -0.04(-0.53,0.44) | 0.856 |
| **3-month** | 2.96±1.93 | 3.35±2.03 | -0.39(-0.89,0.10) | 0.118 |
| **VAS change from baseline to the end of treatment** | -2.78±2.06 | -2.60±2.22 | -0.18(-0.72,0.35) | 0.499 |
| **4-month** | 2.88±1.98 | 3.00±1.96 | -0.12(-0.61,0.38) | 0.646 |
| **5-month** | 3.04±1.99 | 2.79±1.93 | 0.25(-0.24,0.74) | 0.309 |
| **6-month** | 2.83±1.98 | 2.71±1.96 | 0.12(-0.37,0.61) | 0.633 |
| **VAS change from baseline to the end of follow-up** | -2.92±2.27 | -3.25±2.20 | 0.33(-0.23,0.89) | 0.248 |

Missing data were imputed by multiple imputation approach.

The analysis used a *t*-test.

XFZY: Xuefu Zhuyu oral liquid; VAS: visual analogue scale; SD: standard deviation; CI: confidence interval.

**Table S5 Subgroup analyses of primary outcome**

| **Subgroup** | **XFZY** | **Placebo** | **95%CI of Difference** | ***P* for interaction** |
| --- | --- | --- | --- | --- |
| **All Patients** | 126 | 123 | -0.18(-0.72,0.35) | / |
| **Baseline PD Severity （Mean VAS）** |  |  |  |  |
| Mean VAS <7 | 102 | 96 | -0.24 (-0.79, 0.30) | 0.974 |
| Mean VAS ≥7 | 24 | 27 | -0.22 (-1.50, 1.05) |  |
| **Baseline PD Severity (Peak VAS)** |  |  |  |  |
| Highest VAS <7 | 64 | 60 | -0.27 (-0.93, 0.39) | 0.799 |
| Highest VAS≥7 | 62 | 63 | -0.14 (-0.94, 0.67) |  |
| **Baseline Treatment** |  |  |  |  |
| Yes | 45 | 45 | -0.24 (-1.19, 0.72) | 0.886 |
| No | 81 | 78 | -0.15 (-0.80, 0.49) |  |
| **Course of disease (Months)** |  |  |  |  |
| ≤ 60 | 69 | 59 | 0.27 (-0.46, 0.99) | 0.086 |
| > 60 | 57 | 64 | -0.67 (-1.47, 0.13) |  |

Missing data were imputed.

The analysis used a *t*-test.

XFZY: Xuefu Zhuyu oral liquid; VAS: visual analogue scale; CI: confidence interval.

**Table S6 Number of participants with CM syndrome changed**

| **Periods** | **XFZY group**  **(n = 126)** | **Placebo group**  **(n = 123)** | **χ2** | ***P*** |
| --- | --- | --- | --- | --- |
| **3-month (%)** | 11(9.3) | 5(4.2) | 2.414 | 0.120 |
| **6-month (%)** | 20(17.1) | 7(5.9) | 7.197 | 0.007 |

Chi-Square was used.

CM: Chinese medicine; XFZY: Xuefu Zhuyu oral liquid.

**Table S7 Adverse events list.**

| **Adverse event** | **XFZY group**  **(n=129)** | **Placebo group**  **(n=126)** |
| --- | --- | --- |
| **Overall** | 10 | 4 |
| **Constipation** | 1 | 1 |
| **Diarrhea** | 1 | 2 |
| **Abdominal distention** | 0 | 1 |
| **Cold** | 2 | 0 |
| **Cough** | 1 | 0 |
| **Headache** | 3 | 0 |
| **Throat pain** | 2 | 0 |

XFZY: Xuefu Zhuyu oral liquid.
